# Inhibition of Human Cervical Cancer Cell Growth by Ethanolic Extract of *Boerhaavia diffusa* Linn. (Punarnava) Root

**DOI:** 10.1093/ecam/nep223

**Published:** 2011-05-02

**Authors:** Rakhi Srivastava, Daman Saluja, Bilikere S. Dwarakanath, Madhu Chopra

**Affiliations:** ^1^Dr. B. R. Ambedkar Center for Biomedical Research, University of Delhi, Delhi 110007, India; ^2^Institute of Nuclear Medicine and Allied Sciences, Delhi 110054, India

## Abstract

In Indian traditional medicine, *Boerhaavia diffusa* (*punarnava*) roots have been widely used for the treatment of dyspepsia, jaundice, enlargement of spleen, abdominal pain and as an anti-stress agent. Pharmacological evaluation of the crude ethanolic extract of *B. diffusa* roots has been shown to possess antiproliferative and immunomodulatory properties. The extract of *B. diffusa* was studied for anti-proliferative effects on the growth of HeLa cells and for its effect on cell cycle. Bio-assays of extracts from *B. diffusa* root showed that a methanol : chloroform fraction (BDF 5) had an antiproliferative effect on HeLa cells. After 48 h of exposure, this fraction at a concentration of 200 **μ**g mL^−1^ significantly reduced cell proliferation with visible morphological changes in HeLa cells. Cell cycle analysis suggests that antiproliferative effect of BDF 5 could be due to inhibition of DNA synthesis in S-phase of cell cycle in HeLa cells, whereas no significant change in cell cycle was detected in control cells. The fraction BDF 5 caused cell death via apoptosis as evident from DNA fragmentation and caspase-9 activation. Thus the extract has potential to be evaluated in detail to assess the molecular mechanism-mediated anticancer activities of this plant.

## 1. Introduction

Cervical cancer is the most common cancer among women in several regions of India [1]. Of the 500 000 new cases of cervical cancer reported worldwide annually, India accounts for one-fifth in terms of overall incidence [2]. Natural products are the most consistently successful source of pharmacologically active compounds in which plant materials deserves an important position. *Boerhaavia diffusa* L. (Nyctaginaceae), commonly known as “punarnava" in the Indian system of medicine, is a perennial creeping herb found abundantly all over India. In old Indian books of medicine such as the *Charaka Samhita* and *Sushrita Samhita*, it is mentioned that the Ayurvedic preparations made from *punarnava*—namely, *punarnavastaka kvath*, *punarnava kshar* and *punarnava taila*—were used for the treatment of various ailments [3]. In Indian traditional medicine, roots of *B. diffusa* have been widely used for the treatment of dyspepsia, jaundice, enlargement of spleen, abdominal pain and as an anti-stress agent [4, 5]. Pharmacological studies have demonstrated that *punarnava* possesses punarnavoside, which exhibits a wide range of properties—diuretic [6], antifibrinolytic [7], anticonvulsant [8], antibacterial [9]. Scientific studies using the extract of this plant showed that it has got analgesic and anti-inflammatory property [10, 11], hepato-protective activity [12, 13], immunomodulatory activity [14–16] and anti-proliferative properties [17]. Liriodendrin isolated from the methanol extract of the roots of *B. diffusa* was found to exhibit significant calcium channel antagonistic activity [18]. Similarly, methanol extract also exhibited a significant spasmolytic activity in the guinea pig ileum, through a direct effect on the smooth muscle [19]. The aqueous methanol (3 : 7) extract of *B. diffusa* was found to be effective in reducing the metastasis formation by B16F10 melanoma cells [20]. Punarnavine, an alkaloid from *B. diffusa* could enhance the immune response against metastatic progression of B16F-10 melanoma cells in mice [21]. Eupalitin-3-O–*β*-d-galactopyranoside (Bd-1) isolated and purified from the ethanolic leaf extract of *B. diffusa* shows selective immunosuppressive activity [22]. Whole-plant extract of *B. diffusa* has radioprotective effect [23]. Two rotenoids isolated from *B. diffusa*, boeravinones G and H, have been found to potently inhibit the drug efflux activity of breast cancer resistance protein (BCRP/ABCG2), a multidrug transporter responsible for cancer cell resistance to chemotherapy [24]. Most of these studies were either done with crude extract of the plant or with some known isolated compounds. In order to isolate novel lead/active principal from *B. diffusa*, we wanted to explore its antiproliferative action on cervical cancer. So far there is no such report for this plant showing its effect on cervical cancer. Investigations on the chemical constituents of the plant have indicated the occurrence of several rotenoids namely boeravinone A-J [24–28] and two alkaloids, Punarnavine-1 and Punarnavine-2, belonging to the group quinolizidine [29]. Though the compound from the roots, seeds and leaves of *B. diffusa*, isolation of *β*-sitosterol, *β*-sitosterol-*β*-d-glucoside, tetracosanoic, hexacosanoic, stearic, palmitic, arachidic acids, hextriacantane, urosolic acid has been reported [7, 30]; however, it is not known if any of these compounds have antiproliferative and cytotoxic activity. In spite of various therapeutic effects of *B. diffusa*, little is known about the antiproliferative action of root extract of the plant and its detailed mechanism of action. This prompted us to investigate the growth inhibitory effect of this plant on cervical cancer cell line.

## 2. Methods

### 2.1. Plant Material and Extraction Procedures

Herb *B. diffusa* was collected from Gwalior, India, in the month of June 2004, and identified by Dr Gurcharan Singh, Department of Botany, Sri Guru Teg Bahadur Khalsa College, University of Delhi, Delhi. The dried roots of this plant were cut into small pieces and ground into powder. The powder (110 g) was macerated with ether (1 L) and allowed to stand for about 24 h at room temperature. There after the percolate was collected and the process of extraction was repeated six times. After removing the ether extract, the residue was macerated with 95% ethanol (1 L) followed by water (1 l), each for six times. The extracts were filtered before evaporating to dryness under reduced pressure at 45°C with a Rotary evaporator. The percentage yield of the crude extracts was calculated as: (weight of crude extract/weight of fresh plant) × 100%.

### 2.2. Bioactivity-Guided Purification

**Figure 1:**
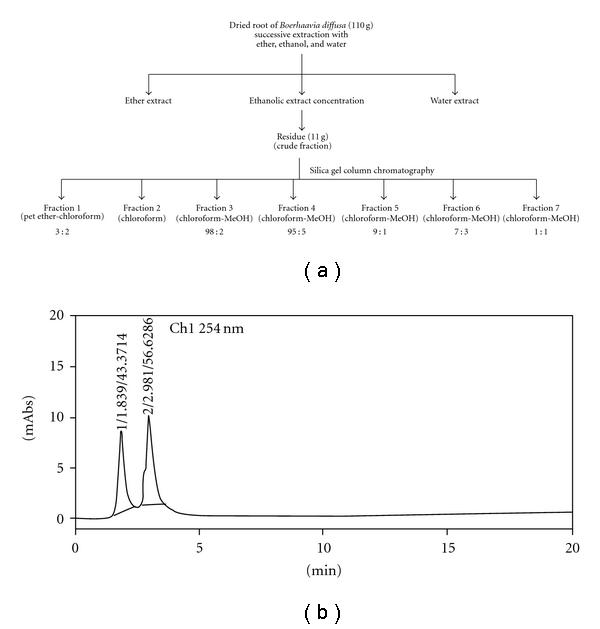
Bioactivity-guided purification. (a) Bioactivity-guided fractionation on silica gel column chromatography of ethanolic root extract. (b) Chromatogram of active fraction (BDF 5) resolved using mobile phase methanol : water (50 : 50) v/v at a flow rate of 1 mL min^−1^.

All extracts obtained from three different extraction solvents (ether, ethanol and water) were subjected to cell proliferation assay. From the bioassay results ethanolic extract, which showed significant inhibitory effect was further subjected for purification using column chromatography. The stationary phase was made up of a glass column packed with silica gel 60–200 mesh size. The mobile phase consisted of combinations of petroleum ether, chloroform and methanol (MeOH), and the eluting strength of the solvent was increased gradually by increasing the composition of the more polar solvent. For purification of the ethanolic extract, the initial solvent composition was petroleum ether (100% v; 300 mL) and then it was changed to petroleum ether: chloroform (4:  1 v/v; 500 mL), followed by petroleum ether : chloroform (3 : 2 v/v; 300 mL), petroleum ether : chloroform (2 : 3 v/v; 200 mL), petroleum ether : chloroform (1 : 4 v/v; 400 mL), chloroform (100% v; 1500 mL), chloroform : MeOH (98 : 2 v/v; 800 mL), chloroform : MeOH (95 : 5 v/v; 1100 mL), chloroform : MeOH (9 : 1 v/v; 1500 mL), chloroform : MeOH (4 : 1 v/v; 800 mL), chloroform : MeOH (7 : 3 v/v; 500 mL), chloroform : MeOH (1 : 1 v/v; 300 mL) and finally to MeOH (100% v; 500 mL). The eluent was collected in fractions of 100 mL each. The chemical composition of each fraction was evaluated by using thin-layer chromatography (TLC) and visualized with UV (254 and 365 nm) and iodine vapors. Based on the TLC profiles, fractions with similar compositions were pooled together and concentrated under reduced pressure. A total of seven major combined fractions were obtained from the ethanol extract. A diagram of the purification process is illustrated in Figure 1(a). 

The fractions obtained through column chromatography were subjected to the antiproliferation assay on HeLa cell line and following that one most active sample (BDF 5) was selected for evaluation of its antiproliferative effect on the HeLa cell line. The HPLC of *B. diffusa* fraction 5 (BDF 5) was performed on the Shimadzu HPLC system using reverse phase C-18 column and UV detector (254 nm). Methanol : water (50 : 50; v/v) was used as the mobile phase and the flow rate was maintained at 1 mL min^−1^.

### 2.3. Sample Preparation of *B. diffus* Extract

*Boerhaavia diffusa* ethanolic root extract and different fractions were dissolved in dimethyl sulfoxide (DMSO, Sigma, St. Louis, USA). For all experiments, final concentrations of the tested compounds were prepared by diluting the stock with the culture medium.

### 2.4. Cell Lines and Culture Medium

HeLa (cervical cancer) and other cell lines like U-87 (human glioma), Hep 3B (hepatic cancer), HCT-15 (colon cancer), NIH 3T3 (mouse embryonic fibroblast) were purchased from NCCS, Pune, and cultured in Dulbecco's modified Eagle medium (Sigma, St. Louis, USA) supplemented with 10% heat inactivated fetal bovine serum (GIBCO) and 1% penicillin-streptomycin (Sigma, St. Louis, USA). The cells were incubated in a humidified atmosphere of 5% CO_2_ at 37°C. All the cell lines used in the study were of passage number between 5 and 10.

### 2.5. *In Vitro* Cytotoxicity Assay

Cell survival was measured using the MTT microculture tetrazolium assay, according to the method described by Mosmann [31] with slight modifications. Briefly, cells at the exponential growth phase were trypsinized and resuspended in the complete medium to a population of 0.25 × 10^5^ cells mL^−1^. A total of 5000 cells per well were seeded in a 96-well plate. After 24 h incubation in a 5% humidified CO_2_ incubator at 37°C, varying concentrations of BD root extract were added to final volume of 200 *μ*l of standard growth medium per well. The concentration of DMSO used to dissolve the extract did not exceed 0.3% (v/v), and therefore the same concentration of DMSO was used in control wells. Methotrexate (at a concentration of 10, 20, 50, 100 and 200 nM) was used as a positive control. After 72 h incubation at 37°C, 20 *μ*L of MTT [3-(4,5-dimethylthiazol-2-yl)-2,5- diphenyl tetrazolium bromide] (Invitrogen) 5 mg mL^−1^ in phosphate buffer saline (PBS) was added to each well and incubated for 4 h at 37°C. The medium was removed and formazan crystals thus formed were dissolved in DMSO. The plates were read immediately in a microplate reader (Tecan, Genios-Pro, Austria) operating at 540 nm.

### 2.6. Cell Proliferation Kinetics

After treatment, cells were incubated in the growth medium for varying intervals of time, harvested by trypsinization and counted in a hemocytometer (adherent and floating cells pooled together) [32]. Cell proliferation was calculated by computing the increase in the cell number and the cell proliferation index *P*, calculated as

(1) $$p=\frac{N_{t}}{N_{0}},$$

where *N*_*t*_: number of cells at time *t* and *N*_0_: number of cells at the time of treatment.

### 2.7. Morphological Analysis

HeLa cells in the exponential growth phase were plated at 2 × 10^4^ cells in a 40 mm Petri plate. After 24 h growth, cells were treated with BDF 5 (200 *μ*g mL^−1^) and vehicle control for different time periods. At the end of the treatment, the effect of BDF 5 on morphological change of the HeLa cells was assessed by the phase-contrast microscope (Nikon, Japan) at 100 × magnification.

### 2.8. Analysis of DNA Fragmentation

In a medium containing 10% FBS, 0.5 × 10^6^ cells were incubated for 24 h. After 24 h, cells were treated with BDF 5 at a concentration of 200 *μ*g mL^−1^. For each experimental time point, cells were collected by trypsinization and rinsed twice in cold phosphate buffered saline (PBS, pH 7.4). Genomic DNA was extracted from HeLa cells as described earlier [33]. Briefly cells were re-suspended twice in a lysis buffer containing 1% Nonidet-P40, 20 mM EDTA and 50 mM Tris-HCl, pH 8. The cells were centrifuged at 1600 g for 10 min, recovered supernatant were combined and incubated with 0.5% SDS and 0.5 mg mL^−1^ RNase A (Sigma, St. Louis, USA) at 56°C for 2 h and thereafter treated with 1 mg mL^−1^ Proteinase K (Bangalore Genei, India) at 37°C for 4 h. The DNA was precipitated by the addition of 1/10 volume of 7.5 M ammonium acetate and two volumes of ethanol and analyzed by agarose gel electrophoresis.

### 2.9. Cell Cycle Analysis

The distribution of cells at different stages in the cell cycle was estimated by flow cytometric DNA analysis. Flow cytometric measurements of cellular DNA content were performed with the ethanol (70%)-fixed cells using the intercalating DNA fluorochrome, propidium iodide as described by Dwarakanath et al. [34]. Briefly, 5 × 10^5^ cells were incubated overnight in 60 mm dishes in a medium containing 10% FBS. After 24 h, cells were treated with BDF 5 at a final concentration of 200 *μ*g mL^−1^. Cells were harvested at different time intervals, washed twice with cold PBS (pH 7.4) and fixed with 70% ethanol/30% PBS at 4°C. The fixed cells were washed with PBS to remove ethanol and incubated with 0.2 mL PBS containing RNase (200 *μ*g mL^−1^) (Sigma, St. Louis, USA) and incubated at 37°C for 30 min, and then stained with 50 *μ*g mL^−1^ propidium iodide (PI, Sigma, St. Louis, MO, USA) for 30 min in the dark at room temperature, and finally analyzed on a FACS cytometer (Calibur, Becton Dickinson, USA). A minimum of 1 × 10^4^ cells per sample was evaluated, and the percentage of cells in each cell cycle phase was calculated using the CELLQUEST and Modfit software (Becton Dickinson).

### 2.10. BrdU Pulse Labeling

BDF 5 treated cells were pulsed with BrdU (a thymidine analogue) (Sigma, St. Louis, USA) at a concentration of 10 *μ*M, 10 min before each time point. After each time point cells were trypsinized and washed with cold PBS twice, fixed with 70% ethanol and stored at 4°C until used. The immunofluorescence staining for flow cytometric analysis was performed as described by Zolzer et al. [35]. Cells were washed with cold saline (0.9% NaCl), cell pellet was incubated with pepsin (0.5% in 0.055 N HCl, pH 1.8) for 10 min at 37°C. Unwinding of DNA was done with 2N HCl for 30 min at room temperature and washed with PBS. The cells were then incubated with first antibody (anti-BrdU 1 : 300 in PBS-Tween) (Santa Cruz Biotechnology, Inc.) for 45 min at 4°C. Non-specific staining is prevented by first blocking with PBS-Tween 20 (0.05%)–BSA (1%) followed by incubation with secondary antibody (1 : 500 in PBS-Tween–BSA) (Santa Cruz Biotechnology, Inc.) for 45 min, at 4°C. Cells were then stained with propidium iodide (50 *μ*g mL^−1^). Cells were finally acquired by a FACScalibur flow cytometer (Calibur, Becton Dickinson, USA) and analysis was performed using ModFit software; 10 000 events were collected, corrected for debris and aggregate populations.

### 2.11. Western Blot Analysis

Following appropriate treatment, cells were detached and collected by centrifugation (600 g, 5 min, 4°C). Whole cell protein was extracted as described earlier [36]. The pellets were washed with 1 mL of ice-cold PBS, collected again via centrifugation (600 g, 5 min, 4°C) and resuspended in a lysis buffer containing 25 mM HEPES, 5 mM MgCl_2_, 2 mM EDTA, 2 mM DTT, 1 mM PMSF, 1 mM sodium orthovanadate, 1% SDS, 1% Triton X-100 and 1% protease inhibitor cocktail (Sigma, St. Louis, USA). Lysates of 5 × 10^6^ cells were sonicated and then centrifuged (18 000 g, 10 min, 4°C). After sonication, lysates were centrifuged as above, and the supernatant was collected.

Equal amounts of protein (50 *μ*g), as determined using the Bradford Protein estimation kit (GeNei, Bangalore Genei, India), were loaded and resolved using 12% SDS-PAGE and transferred onto PVDF membranes (Mdi, Ambala, India). Blots were blocked overnight at 4°C in PBS-Tween 20 (0.05%)–BSA (3%) and then incubated with primary antibody in the blocking buffer (overnight, 4°C). The antibodies used in this study included caspase-3, caspase-9 and anti-*β*-actin. All primary antibodies used were obtained from Santa Cruz Biotechnology (Santa Cruz, CA, USA). After washing with PBS containing 0.05% Tween (PBS-T), blots were incubated with secondary antibody; goat-anti-mouse IgG-HRP for caspase-9 and donkey-anti-goat IgG-HRP for caspase-3 and *β*-actin (Santa Cruz, CA, USA) for 2 h at 4°C. Following successive washes, the blots were developed using the DAB system (GeNei, Bangalore Genei, India). Photographs were taken using GeneSnap acquisition software (Syngene, Cambridge, UK). GeneTools analysis software was used for the quantification of the bands.

### 2.12. Statistical Analysis

The experiments were performed in triplicate and all experimental data were expressed as mean ± SD. The statistical significance of the difference between control and BD extract-treated groups was determined by one-way ANOVA followed by Dunnett's *t*-tests for multiple comparisons and Student's *t*-test for dual comparison. The results were considered significant at *P* < .05.

## 3. Results

### 3.1. Growth Inhibition of Human Cancer Cell Lines by the Extracts from *B. diffusa*

**Figure 2:**
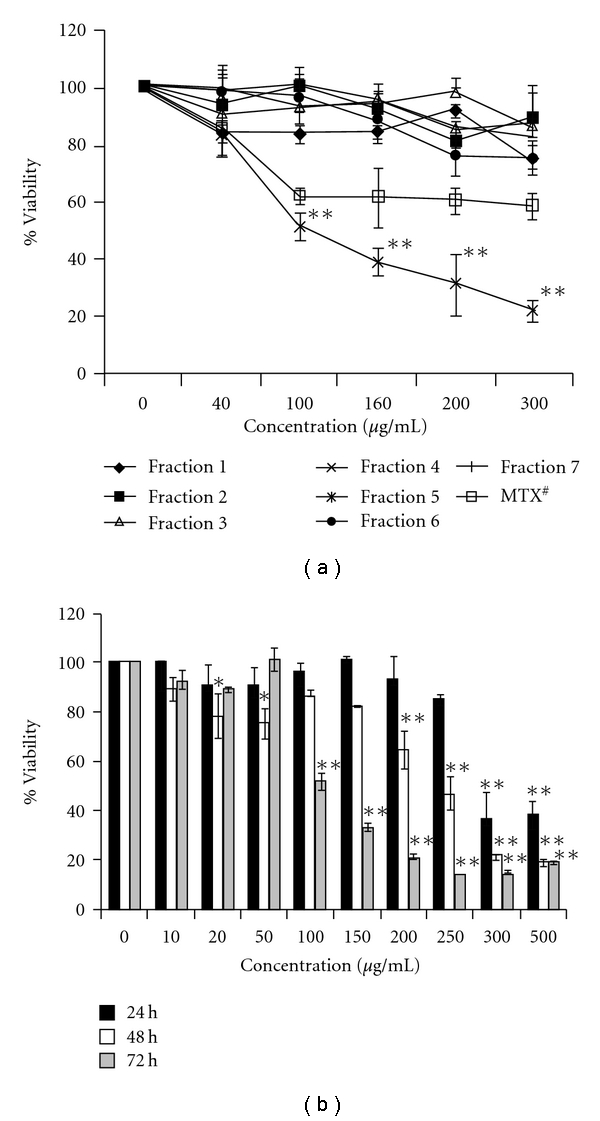
Effects of different fractions of BD extract on HeLa cells. (a) Cultured cells were exposed to various concentrations of different fractions for 72 h. Cell viability was analyzed by MTT assay. ^#^Methotrexate was at a concentration of 10, 20, 50, 100 and 200 nM (b) Effects of BDF 5 on HeLa cells in time- and dose-dependent manner. Cultured cells were exposed to various concentrations of BDF 5 for different time point. Cell viability was analyzed by MTT assay. The result represents the average of three independent experiments in triplicate ± SD. **P < .01, *P < .05.

Cell lines of different origin might exhibit different sensitivities toward the same compound. Therefore, it was necessary to consider more than one cell line in the initial screening experiment. Bearing this in mind, four cell lines of human origin, namely HeLa (cervical cancer), U-87 (glioma), Hep 3B (hepatic cancer), HCT-15 (colon cancer) and one mouse NIH 3T3 (mouse embryonic fibroblast), were used in the present study. *In vitro* screening of the extract *B. diffusa* indicated that ethanolic crude root extract is cytotoxic against the HeLa cell line. Whereas other cell lines namely U-87, HCT-15, Hep 3B and non-cancerous NIH 3T3 were less sensitive to treatment by BD EtOH crude root extract (data not shown). At a concentration of 300 *μ*g mL^−1^, the crude root extract caused 30% cell death in HeLa cell line. The crude extract was then purified by column chromatography using silica gel as stationary phase and increasing the solvent polarity as given schematically in Figure 1(a). The fractions obtained through column chromatography were subjected to antiproliferation assay on HeLa cell line and following that, one most active sample (BDF 5) was selected for further evaluation of its antiprolirerative effect on this cell line. Fraction 5 (BDF 5) showed maximum cytotoxicity with 85% cell death at 300 *μ*g mL^−1^ in 72 h. The HPLC profile of BDF 5 using a mobile phase (50% methanol : 50% water) revealed two peaks (Figure 1(b)). Characterizations of both of these peaks are currently under investigation. All other fractions did not show significant antiproliferative effect (Figure 2(a)). The partially purified root extract column fraction BDF 5 was tested in a time- as well as dose-dependent manner for the cytotoxic effects on HeLa cell line. BDF 5 causes 55% cell death at a concentration of 300 *μ*g mL^−1^ after exposure for 24 h, while a significant effect was not observed at a concentration of 100 *μ*g mL^−1^. Prolonged treatment (72 h) with BDF 5 shows 85% cell death at a concentration of 300 *μ*g mL^−1^ and 64% cell death even at a 100 *μ*g mL^−1^ concentration (Figure 2(b)). 

### 3.2. Cell Proliferation

**Figure 3:**
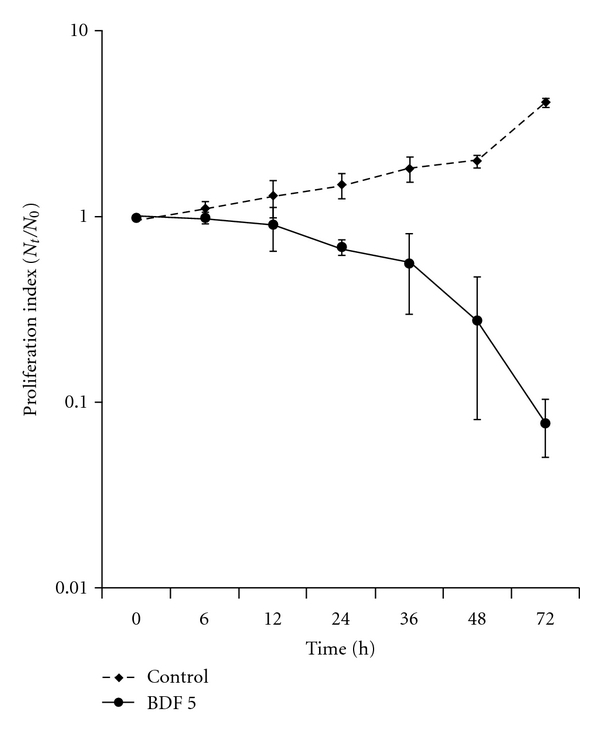
Effect of BDF 5 on growth kinetics of HeLa cells. HeLa cells (5 × 10^5^ cells) in a 60 mm culture plate were exposed to BDF 5 (200 *μ*g mL^−1^) for different time points. Cell number was measured with trypan blue. The result represents the average of three independent experiments in triplicate ± SD.

The effects of BDF 5 on the proliferation of exponentially growing HeLa cells were studied by monitoring the kinetics of cell growth. A time-dependent reduction in the rate of cell proliferation was observed (Figure 3), with a lag period followed by the cytostatic effect up to 12 h and growth inhibition after 24 h treatment. However, almost >50% cells were dead or degenerating at a concentration of 200 *μ*g mL^−1^ at 48 h. 

### 3.3. BDF 5 Causes Visible Morphological Changes of HeLa cells

**Figure 4:**
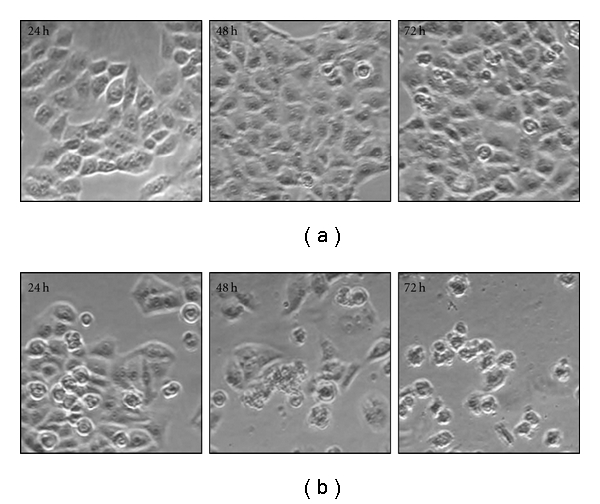
Effect of BDF 5 on morphology of the HeLa cells. Morphological changes of cells were examined under phase contrast microscope at 100x magnification. (a) Vehicle control cells, (b) BDF 5 (200 *μ*g mL^−1^)-treated cells.

We examined morphological changes in the cells in detail using a phase-contrast microscope. The cells underwent marked morphologic changes such as shrinkage, rounding, detachment and membrane blebbing in HeLa cells exposed to 200 *μ*g mL^−1^ of BDF 5 for different time period (Figure 4). These morphological changes suggested that BDF 5 may induce apoptotic cell death in HeLa cells. 

### 3.4. Effect of BDF 5 on DNA Fragmentation

**Figure 5:**
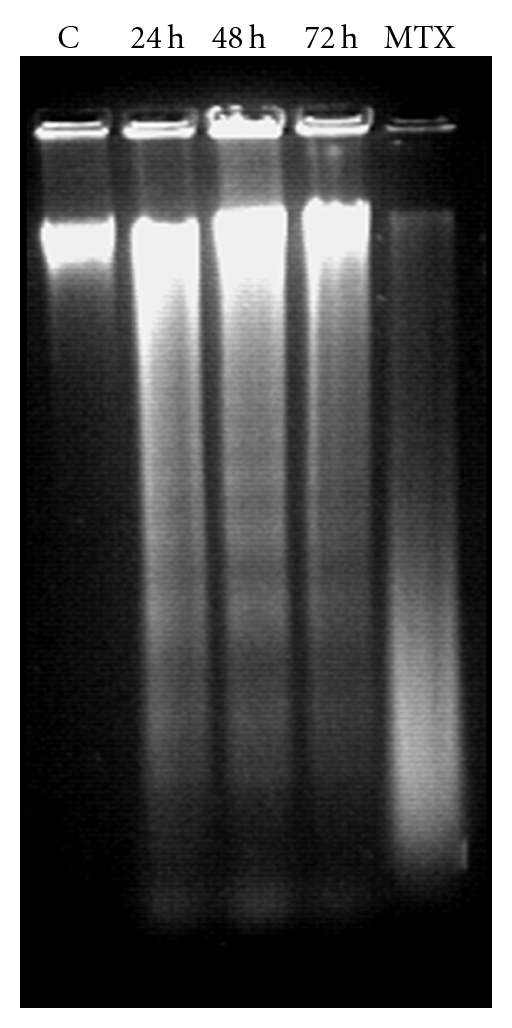
DNA fragmentation in BDF 5-treated HeLa cells. Genomic DNA was extracted from DMSO (0.3%) and BDF 5 (200 *μ*g mL^−1^)-treated HeLa cells after incubation for 24, 48 or 72 h. Nucleosomal DNA fragments were resolved by electrophoresis in a 1.5% agarose gel and visualized by ethidium bromide staining. C, DMSO control; MTX, methotrexate at a concentration of 200 nM.

DNA fragmentation is a characteristic feature of apoptosis [37]. Therefore, BDF 5-induced apoptosis was confirmed by the DNA fragmentation assay. Increased DNA fragmentation was apparent in HeLa cells after treatment with 200 *μ*g mL^−1^ of BDF 5 for 24, 48 and 72 h. A typical experimental result of agarose gel electrophoresis is shown in Figure 5, where the effect of BDF 5 for 72 h treatment produced DNA fragmentation. Whereas treatment with DMSO (0.3%) (negative control) did not produce DNA fragment ladders after 72 h in HeLa cells. Treatment with methotrexate (positive control) at a 200 nM concentration also produced DNA fragment ladders after 72 h treatment in HeLa cells. 

### 3.5. Cell Cycle Perturbations

**Figure 6:**
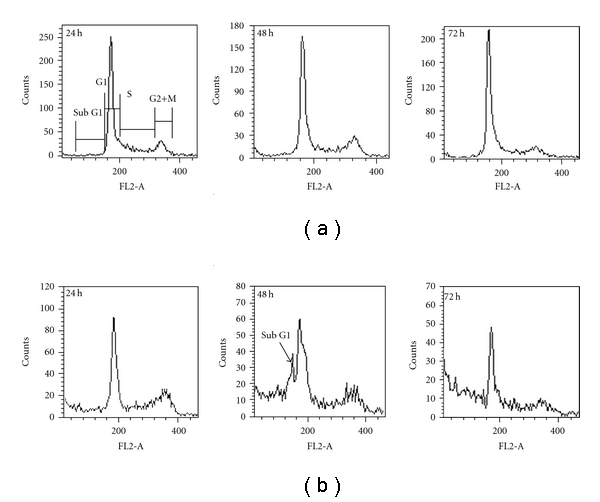
Cell cycle analysis of HeLa cells after treatment with vehicle control (0.3% DMSO) or 200 *μ*g mL^−1^ BDF 5. Cells were collected and processed for cytometric analysis of cell cycle distribution. DNA content was analyzed using PI staining and DNA flow cytometry. (a) Cells were treated with vehicle (0.3% DMSO). (b) Cells were treated with BDF 5 (200 *μ*g mL^−1^) for different time points.

The effect of partially purified fraction on cell cycle progression of HeLa cells was determined by flow cytometry. HeLa cells treated with BDF 5 at a final concentration of 200 *μ*g mL^−1^ showed decrease in G1 phase cells from 52 ± 1.4% to 48 ± 1.2% and this decrease in the G1 phase was accompanied by increase in the population of the G2+M phase from 13 ± 1.5% in control to 16 ± 2.0% in treated cells. BDF 5 also showed moderate inhibition in the progression through the S-phase, with a slight decrease in the population of the S-phase from 30 ± 0.5% to 28 ± 2.5% at 48 h. This was accompanied by an increase in the population of the G2+M phase from 10 ± 2.0% in control to 18 ± 2.0% in the treated cultures. After 72 h, treatment cells get accumulated in the S-phase as well as there is increase in the population of G2+M cells. This was accompanied by a decrease in the proportion of cells in the G1 phase of cell cycle (Table 1). A representative histogram for the HeLa cells is shown in Figure 6. 

From cell cycle analysis we observed the appearance of a peak corresponding to a population of cells with sub-G1 DNA content after 48 h treatment. The DNA histograms revealed a hypo-diploid population after 48 h treatment (Figure 6), suggestive of apoptotic cell death under these conditions.

### 3.6. Determination of S-Phase Cells Using Bromodeoxy Uridine

**Figure 7:**
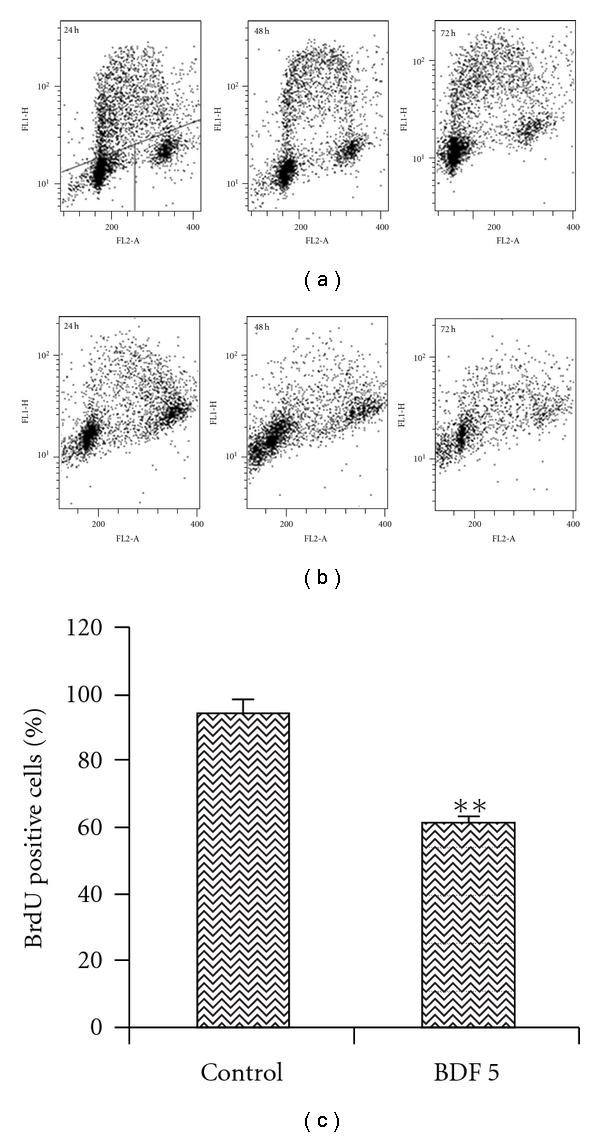
DNA synthesis in untreated HeLa cells and cells treated with BDF 5. Cells were treated with BDF 5 (200 *μ*g mL^−1^) for different time point and pulsed with BrdU. Incorporation of BrdU was detected by immunofluorescence using a BrdU monoclonal antibody. The DNA content, measured by propidium iodide staining of cells, is represented as a linear scale on the abscissa, showing cells with 2 N or 4 N, respectively, in G1 (lower left of each panel) and G2/M (lower right of each panel). The ordinate is a logarithmic scale representing cells in S-phase, based on incorporation of 5-bromodeoxyuridine. (a) Cells were treated with vehicle (0.3% DMSO). (b) Cells were treated with BDF 5 (200 *μ*g mL^−1^) for different time points. (c) Graphical representation of BrdU positive cells in control and BDF 5-treated cells. The data are expressed as mean ± SD from three independent experiments. **P < .01.

To investigate the cells in the S-phase of cell cycle BrdU pulse labeling was performed, the inhibitory effect of fraction 5 (BDF 5) on HeLa cell line was further confirmed using BrdU incorporation into the untreated and treated cells *in vitro.* The effect of BDF 5 on DNA synthesis was thus investigated by measuring BrdU incorporaton by flow cytometry with an anti-BrdU primary monoclonal antibody. Biparametric histograms of BrdU-FITC fluorescence verses PI fluorescence is shown in Figure 7. BrdU-labeled cells in the untreated HeLa cells was 94.09 ± 3.97%, while after 48 h treatment with 200 *μ*g mL^−1^ of BDF 5, BrdU-labeled cells were 60.75 ± 1.88%. This comes out to be 17% of a total 28% S-phase cells as determined by cell cycle analysis. Statistical significances were found in untreated and treated cells (*P* < .01). 

### 3.7. BDF 5-Induced Differential Caspases Proteins Expression Related to Apoptosis in HeLa Cells

**Figure 8:**
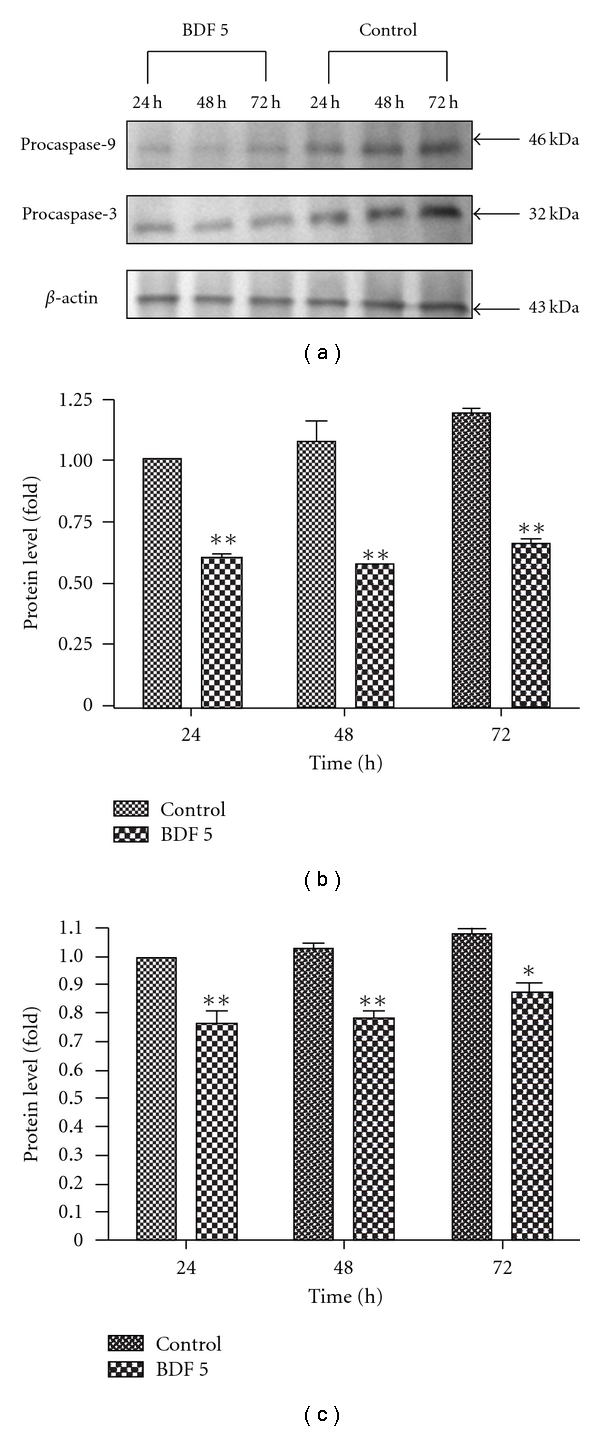
BDF 5-induced apoptotic cell death in HeLa cells. Culture cells were treated with vehicle control (0.3% DMSO) or with BDF 5 at a concentration of 200 *μ*g mL^−1^ for different time periods. After incubation the cells were lysed and the cellular proteins were separated by SDS-polyacrylamide gels and transferred onto PVDF membranes. Procaspase-9 (46 kDa) and procaspase-3 (32 kDa)-specific bands were detected by western blot. Immunoblot represents the observations from one single experiment repeated three times (a). The integrated optical densities (IOD) of caspase-9 and -3 proteins after normalization with *β*-actin (43 kDa) in each lane using GeneTools analysis software (Syngene) were demonstrated in (b) and (c), respectively. Each data point in the figure represents the mean ± SD of three separate experiments. Statistical difference compared to control. *P < .05, **P < .01.

In order to assess the mechanism of BDF 5-induced apoptosis, we evaluated the expressions of caspases by the western blot analysis. Caspases are cytosolic proteins that exist normally as inactive precursors with higher molecular weight (46, 32 kDa). They are cleaved proteolytically into low molecular weights (20–23 kDa) when cell undergo apoptosis [38]. In this study, the expressions of the inactive form of caspase-3 and caspase-9 declined after treatment with BDF 5 (200 *μ*g mL^−1^) at different time periods (Figure 8(a)). Approximately 50% fall in the expression of procaspase-9 and 25% fall in the expression of procaspase-3 in comparison to control after 48 h treatment (Figures 8(b) and 8(c)). 

## 4. Discussion

The treatment of cancer may benefit from the introduction of novel therapies derived from natural products. Natural products have served to provide a basis for many of the pharmaceutical agents in current use in cancer therapy [39]. The root, leaves and aerial parts or the whole plant of *B. diffusa* have been employed for the treatment of various disorders in the Ayurvedic herbal medicine. It was evidenced that the leaves and root possessed antifibrinolitic and anti-inflammatory activities [11]. Ethanolic extracts were normally used for anticancer screening because circumstantial evidences from traditional practitioners believed that mostly the polar compounds are responsible for the claimed anticancer properties. The study, by Mehrotra et al. reported that the ethanolic extract of *B. diffusa* showed a significant antiproliferative and immunosuppressive activity [17]. Taking lead from these we have started our work with ethanolic extract. Leyon et al. showed the inhibitory effect of *B. diffusa* on experimental metastasis [20]. The present study was conducted to study the cytotoxicity activity of BD EtOH root extract as well as its purified fraction (BDF 5) on HeLa cell line to provide an introductory approach for the evaluation of its traditional preparation in order to scientifically validate the therapeutic preparation of this plant in the control of cancer. To the best of our knowledge, this is the first report that analyzes the inhibitory effect of BDF 5 on the S-phase of cell cycle in HeLa cell line. Cell lines of different histological origin might exhibit different sensitivities toward an antiproliferative compound. Cell type antiproliferative specificity is observed in *B. diffusa* ethanolic crude root extract. It showed cytotoxicity against the HeLa cell line and was less toxic to the other cell lines tested. This specificity of plant extracts is likely to be due to the presence of different classes of compounds in the extract, as it has been documented in the case of known classes of compounds [40]. The plant ethanolic extract was fractionated on silica gel column chromatography, according to the compound polarity. These were subjected to the MTT test for antiproliferative activity (Figure 2(a)). The BDF 5 showed the cytotoxicity against the HeLa cell line with the IC_50_ values of 250 *μ*g mL^−1^ at 48 h (Figure 2(b)). Such observation demonstrated that some active components of this plant should be in the chloroform : methanol fraction. However, the antiproliferative activities of this plant might be possibly dependent on cell types including the culture conditions. In this study, the results of cell viability assay and morphological analyses showed that BDF 5 significantly inhibited HeLa cells proliferation in a time-dependent manner (Figures 3 and 4). The antiproliferative activity of BDF 5 on HeLa cells might result, at least in part, from inhibition of DNA synthesis and proliferation, and from induction of apoptosis (Figure 6). Observations from flow cytometric DNA analysis suggests that the mode of cell death induced by BDF 5 is mainly through apoptosis, culminating in secondary necrosis. This has been further strengthened by the DNA fragmentation assay (chromatin fragmentation by internucleosomal DNA cleavage), which is considered a hallmark of apoptosis. The appearance of a DNA ladder was investigated by agarose gel electrophoresis of genomic DNA extracted from DMSO (0.3%)—or BDF 5 (200 *μ*g mL^−1^)-treated HeLa cells. BDF 5-treated cells showed typical internucleosomal DNA fragmentation or “ladder" formation at all time points tested (Figure 5). Cell proliferation kinetics clearly showed that 200 *μ*g mL^−1^ retarded the rate of progression through the S-phase and induced an accumulation of cells in G2+M (Table 1). Although, retarded, it appears that the completion of the S-phase was achievable, but the persistence of the lesions probably prevented cells from undergoing mitosis, thus blocking the cells at G2–M transition through G2 checkpoint control. Inhibition of DNA synthesis and therefore progression through the cell cycle of HeLa cells was confirmed by the reduced BrdU incorporation that correlated well with decreased cell proliferation. BrdU is a thymidine analog that is incorporated in place of thymidine into synthesized DNA strands of actively proliferating cells. Incorporation of BrdU is therefore used as evidence of DNA replication. The ability of BDF 5 to reduce the incorporation of BrdU into DNA can thus be interpreted as the ability of BDF 5 to inhibit DNA synthesis. After treatment with 200 *μ*g mL^−1^ BDF 5 for 48 h, proliferation of BrdU-labeled cells was reduced significantly (*P* < .01) (Figure 7), which indicated that BDF 5 extract affected the proliferation of cervical cancer cells through inhibition of DNA synthesis. Although treatment with BDF 5 did not significantly alter the fraction of S-phase cells as analyzed by DNA analysis (Table 1), it did not rule out the inhibitory effect of BDF 5 on the progression of DNA synthesis (chain elongation) as analysis of DNA content alone does not discriminate between cells in S-phase (based on DNA content) that are actively synthesizing DNA (S +ve) from quiescent S-phase cells (not engaged in DNA synthesis; S −ve or S_0_ cells). Treatments like certain chemotherapeutic drugs and high doses of radiation are well known to arrest cells also in the S-phase and thereby result in growth inhibition. It is possible to discriminate the S +ve from S −ve only with the help of probes that identify cells actively synthesizing DNA; for example the incorporation of radiolabeled thymidine (and counting the radioactivity) or BrdU coupled with anti-BrdU antibody and analysis by flow cytometry. We chose to use the BrdU method. The BrdU pulse labeling experiment measures the fraction of cells which are actively involved in DNA synthesis out of the total S-phase cells. Cell cycle analysis revealed that 48 h after treatment with BDF 5, 28% of the total cells were in the S-phase (as compared to 30% in untreated cultures). Nearly 60% of these S-phase cells (i.e., *∼*17% of the total cells) tested +ve to BrdU incorporation (S +ve cells) suggesting that they were actively involved in DNA synthesis, while the remaining 40% (i.e., 11% of the total population) were quiescent in S-phase (S −ve).

The activation of cystein aspartic-specific proteases (caspases) is commonly thought to be one of the earliest points in the no-return pathway of apoptosis. Caspases are broadly categorized into upstream regulatory caspases and downstream effector caspases [41]. The upstream caspases, such as caspase-8 (death receptor pathway) and caspase-9 (mitochondria pathway), typically have a long N-terminal prodomain that facilitates interaction with and recruitment of proapoptotic proteins, including other caspases [42]. The downstream caspases, such as caspase-3, -6 and -7, typically have short prodomains that primarily cleave protein, which is important for cellular functions, and results in cell apoptosis [43, 44]. Our results indicated that apoptotic signaling triggered by BDF 5 is mainly related to the mitochondrial pathway. We determined whether caspase-9 and caspase-3 might be activated during the induction of apoptosis by BDF 5 because caspase-3 is known to play an essential role as an executor in apoptosis. We observed decrease in the expression level of a 46 kDa precursor (procaspase-9) and a 32-kDa precursor (procaspase-3) after treatment indicating that caspase-9 and caspase-3 were activated by BDF 5 (Figure 8). These results demonstrate conclusively that BDF 5 induces apoptosis in HeLa cells, accompanying by the triggered caspase-9 and caspase-3 activation.

Therefore, it could be concluded that the anti-proliferative activity of BDF 5 might result, at least in part, from inhibition of DNA synthesis, retardation of cell proliferation and induction of apoptotic death of cancer cells. Many anticancer drugs inhibit cell cycle progression. These are exemplified by compounds that (i) interfere with nucleotide metabolism, such as methotrexate, which inhibits dihydrofolate reductase; (ii) damage the DNA, for instance, anthracyclins, topoisomerase inhibitors, or alkylating agents; or (iii) prevent the formation of a functional mitotic spindle, such as the taxanes or vinca alkaloids. All of these perturbations cause some kind of damage to the cell, leading to the activation of specific checkpoints that trigger cell death [45]. Various drugs [1-*β*-d-arabinofuranosylcytosine (ara-C), hydroxyurea (HU), 5-hydroxy-2-formylpyridine thiosemicarbazone (5-HP) and camptothecin sodium salt (camptothecin)] considered to markedly inhibit DNA synthesis and are maximally cytotoxic to cells in the S-phase [46]. A phase-specific agent will be maximally effective only if it allows cycling cells to enter the cytotoxic phase. Thus, a S-phase-specific agent that blocks the progression of G1 cells into S will kill only those cells that are in S at the time the drug is added. Sinclair showed that HU2 has such an effect, namely the cells in the S-phase are killed, while the non-S-phase cells accumulate at the G1-S boundary [47, 48]. When HU is removed, the accumulated cells proceed synchronously through the cell cycle. Ara-C has been shown to have similar effects [49]. Inhibition of DNA synthesis could be caused by incorporation of the molecule into DNA, as is the case for Ara-C and dFdC. More likely, it is possible that BDF 5 directly inhibits DNA polymerases or binds to DNA in a non-intercalative mode, thereby interfering with DNA chain elongation. These possibilities are currently under investigation.

## 5. Conclusion

**Figure 9:**
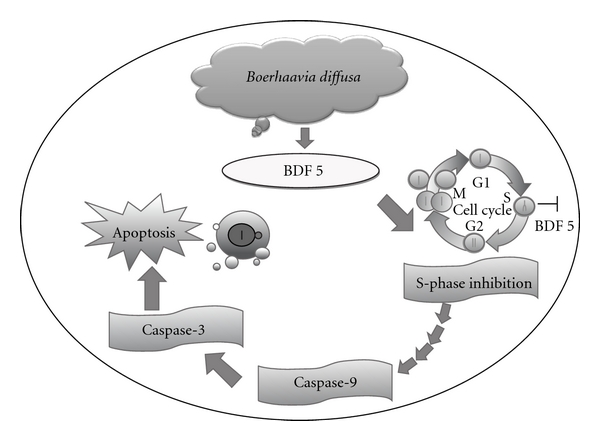
An overview of apoptotic effects elicited by *B. diffusa* fraction 5 (BDF 5) on HeLa cells. S-phase inhibition plays some roles in BDF 5-induced antiproliferative activities. The apoptotic mechanism was mediated by the activation of caspase-9 (upstream regulatory caspase) and caspase-3 (downstream effector caspase). This was subsequently resulting in biochemical and morphological alterations, including DNA fragmentation, membrane blebbing and formation of apoptotic bodies.

In conclusion, *B. diffusa* fraction 5 (BDF 5) could inhibit the proliferation of human cervical cancer cell line, HeLa. Our results demonstrated that the cell cycle via S-phase inhibition plays some roles in *B. diffusa-*induced antiproliferative activities in the HeLa cell line. Taken together, the findings of this study are schematically presented in Figure 9. The alcoholic and water extracts of *B. diffusa* is known to contain several bioactive molecules such as reducing sugars, starch and lignans liriodendrin and syrigaresinol. Several Boeravionones (Boeravionones A–J, etc.) have also been isolated from *B. diffusa*. The activity shown by the partially purified fraction 5 (BDF 5) may be attributed to these diverse compounds. Although scientific studies have been done on a large number of Indian botanicals, a considerably smaller number of marketable drugs of phytochemical entities have entered the evidence-based therapeutics [50]. The authors would like to ascertain that *B. diffusa* is a promising drug entity which should enter the world market by evidence-based research for therapeutics. However, further biochemical work and investigations at the molecular level are currently under progress in our laboratory to identify the active components that could induce growth inhibition and to establish the possible explanation of mechanism of DNA synthesis inhibition by the herb extract. 

## Funding

This work was supported by grants from Indian Council of Medical Research; New Delhi.

## Figures and Tables

**Table 1:** Cell cycle distribution of HeLa cell line after treatment with vehicle control (DMSO, 0.3%) or BDF 5 (200 *μ*g mL^−1^).

Distribution (% cells)	Control cells			BDF 5-treated Cells		
	24 h	48 h	72 h	24 h	48 h	72 h
G1	52 ± 1.4	60 ± 1.5	59 ± 1.5	48 ± 1.2	54 ± 0.5	47 ± 1.1
G2+M	13 ± 1.5	10 ± 2.1	9 ± 0.6	16 ± 2.0	18 ± 2.1	14 ± 2.0
S	35 ± 1.6	30 ± 0.5	32 ± 2.0	36 ± 1.8	28 ± 2.5	39 ± 3.1
Sub-G1	1.8 ± 0.3	2.4 ± 0.5	2.4 ± 0.1	15.5 ± 3.5	25 ± 4.0	34 ± 4.1

The data are expressed as mean ± SE from four independent experiments.

## References

[1] Sharma DC (2001). India favours acetic acid for early detection of cervical cancer. *The Lancet Oncology*.

[2] Sridhar N (2001). New initiatives to combat cervical cancer in India. *The Lancet Infectious Diseases*.

[3] Sahu AN, Damiki L, Nilanjan G, Dubey S (2008). Phytopharmacological review of *Boerhaavia diffusa* Linn. (Punarnava). *Pharmacognosy Reviews*.

[4] Kirtikar KR, Basu BD (1956). *Indian Medicinal Plants*.

[5] Chopra RN, Nayar SL, Chopra IC (1996). *Glossary of Indian Medicinal Plants*.

[6] Gaitonde BB, Kulkarni HJ, Nabar SD (1974). Diuretic activity of punarnava (*Boerhaavia diffusa*). *Bulletin of Haffkine Institute*.

[7] Jain GK, Khanna NM (1989). Punarnavoside: a new antifibrinolytic agent from *Boerhaavia diffusa* Linn. *Indian Journal of Chemistry*.

[8] Adesina SK (1979). Anticonvulsant properties of the roots of *Boerhaavia diffusa*. *Quarterly Journal of Crude Drug Research*.

[9] Olukoya DK, Idika N, Odugbemi T (1993). Antibacterial activity of some medicinal plants from Nigeria. *Journal of Ethnopharmacology*.

[10] Bhalla TN, Gupta MB, Sheth PK, Bhargava KP (1968). Antiinflammatory activity of *Boerhaavia diffusa*. *Indian Journal of Physiology and Pharmacology*.

[11] Hiruma-Lima CA, Gracioso JS, Bighetti EJ, Germonsen Robineou L, Souza Brito AR (2000). The juice of fresh leaves of *Boerhaavia diffusa* L. (Nyctaginaceae) markedly reduces pain in mice. *Journal of Ethnopharmacology*.

[12] Rawat AKS, Mehrotra S, Tripathi SC, Shome U (1997). Hepatoprotective activity of *Boerhaavia diffusa* L. roots—a popular Indian ethnomedicine. *Journal of Ethnopharmacology*.

[13] Chandan BK, Sharma AK, Anand KK (1991). *Boerhaavia diffusa*: a study of its hepatoprotective activity. *Journal of Ethnopharmacology*.

[14] Mungantiwar AA, Nair AM, Saraf MN (1997). Adaptogenic activity of aqueous extract of the roots of *Boerhavia diffusa* Linn. *Indian Drugs*.

[15] Mungantiwar AA, Nair AM, Shinde UA (1999). Studies on the immunomodulatory effects of *Boerhaavia diffusa* alkaloidal fraction. *Journal of Ethnopharmacology*.

[16] Mehrotra S, Mishra KP, Maurya R, Srimal RC, Singh VK (2002). Immunomodulation by ethanolic extract of *Boerhaavia diffusa* roots. *International Immunopharmacology*.

[17] Mehrotra S, Singh VK, Agarwal SS, Maurya R, Srimal RC (2002). Antilymphoproliferative activity of ethanolic extract of *Boerhaavia diffusa* roots. *Experimental and Molecular Pathology*.

[18] Lami N, Kadota S, Kikuchi T, Momose Y (1991). Constituents of the roots of *Boerhaavia diffusa L*. III. Identification of Ca^2+^ channel antagonistic compound from the methanol extract. *Chemical & Pharmaceutical Bulletin*.

[19] Borrelli F, Ascione V, Capasso R, Izzo AA, Fattorusso E, Taglialatela-Scafati O (2006). Spasmolytic effects of nonprenylated rotenoid constituents of *Boerhaavia diffusa* roots. *Journal of Natural Products*.

[20] Leyon PV, Lini CC, Kuttan G (2005). Inhibitory effect of *Boerhaavia diffusa* on experimental metastasis by B16F10 melanoma in C57BL/6 mice. *Life Sciences*.

[21] Manu KA, Kuttan G (2007). Effect of punarnavine, an alkaloid from *Boerhaavia diffusa*, on cell-mediated immune responses and TIMP-1 in B16F-10 metastatic melanoma-bearing mice. *Immunopharmacology and Immunotoxicology*.

[22] Pandey R, Maurya R, Singh G, Sathiamoorthy B, Naik S (2005). Immunosuppressive properties of flavonoids isolated from *Boerhaavia diffusa* Linn. *International Immunopharmacology*.

[23] Manu KA, Leyon PV, Kuttan G (2007). Studies on the protective effects of *Boerhaavia diffusa* L. against gamma radiation-induced damage in mice. *Integrative Cancer Therapies*.

[24] Ahmed-Belkacem A, Macalou S, Borrelli F (2007). Nonprenylated rotenoids, a new class of potent breast cancer resistance protein inhibitors. *Journal of Medicinal Chemistry*.

[25] Kadota S, Lami N, Tezuka Y, Kikuchi T (1988). Structure and NMR spectra of boeravinone C, a new rotenoid analogue from *Boerhaavia diffusa* Linn. *Chemical & Pharmaceutical Bulletin*.

[26] Kadota S, Lami N, Tezuka Y, Kikuchi T (1989). Constituents of the roots of *Boerhaavia diffusa* L. Examination of sterols and structure of new rotenoids, boeravinones A and B. *Chemical & Pharmaceutical Bulletin*.

[27] Lami N, Kadota S, Kikuchi T (1991). Constituents of the roots of *Boerhaavia diffusa* L. IV. Isolation and structure determination of boeravinones D, E, and F. *Chemical & Pharmaceutical Bulletin*.

[28] Borrelli F, Milic N, Ascione V (2005). Isolation of new rotenoids from *Boerhaavia diffusa* and evaluation of their effect on intestinal motility. *Planta Medica*.

[29] Nandi RP, Chatterjee SK (1974). Occurrence of punarnavines in *Boerhaavia repens* Linn. *Indian Journal of Experimental Biology*.

[30] Misra AN, Tiwari HP (1971). Constituents of roots of *Boerhaavia diffusa*. *Phytochemistry*.

[31] Mosmann T (1983). Rapid colorimetric assay for cellular growth and survival: application to proliferation and cytotoxicity assays. *Journal of Immunological Methods*.

[32] Singh S, Dwarakanath BS, Mathew TL (2004). DNA ligand Hoechst-33342 enhances UV induced cytotoxicity in human glioma cell lines. *Journal of Photochemistry and Photobiology B*.

[33] Negri C, Bernardi R, Donzelli M, Scovassi AI (1995). Induction of apoptotic cell death by DNA topoisomerase II inhibitors. *Biochimie*.

[34] Dwarakanath BS, Adhikari JS, Jain V (1999). Hematoporphyrin derivatives potentiate the radiosensitizing effects of 2-deoxy-D-glucose in cancer cells. *International Journal of Radiation Oncology Biology Physics*.

[35] Zolzer F, Hillebrandt S, Streffer C (1995). Radiation induced G1- block and p53 status in six human tumor cell lines. *Radiotherapy & Oncology*.

[36] Shacka JJ, Sahawneh MA, Gonzalez JD, Ye Y-Z, D’Alessandro TL, Estévez AG (2006). Two distinct signaling pathways regulate peroxynitrite-induced apoptosis in PC12 cells. *Cell Death and Differentiation*.

[37] Allen RT, Hunter WJ, Agrawal DK (1997). Morphological and biochemical characterization and analysis of apoptosis. *Journal of Pharmacological and Toxicological Methods*.

[38] Solary E, Plenchette S, Sordet O (2001). Modulation of apoptotic pathways triggered by cytotoxic agents. *Therapie*.

[39] Pietras RJ, Weinberg OK (2005). Antiangiogenic steroids in human cancer therapy. *Evidence-Based Complementary and Alternative Medicine*.

[40] Cragg GM, Boyd MR, Cardellina JH, Newman DJ, Snader KM, McCloud TG, Chadwick DJ, Marsh J (1994). Ethnobotany and drug discovery: the experience of the US National Cancer Institute. *Ethnobotany and Search for New Drugs*.

[41] Cryns V, Yuan J (1998). Proteases to die for. *Genes and Development*.

[42] Ashkenazi A, Dixit VM (1998). Death receptors: signaling and modulation. *Science*.

[43] Gupta S (2001). Molecular steps of death receptor and mitochondrial pathways of apoptosis. *Life Sciences*.

[44] Srinivasula SM, Ahmad M, MacFarlane M (1998). Generation of constitutively active recombinant caspases-3 and -6 by rearrangement of their subunits. *Journal of Biological Chemistry*.

[45] Tatjana V, Achenbach EP, Slater HB, Thorsten B, Rolf M (2000). Inhibition of cyclin-dependent kinase activity and induction of apoptosis by preussin in human tumor cells. *Antimicrob Agents Chemother*.

[46] Bhuyan BK, Fraser TJ, Gray LG, Kuentzel SL, Neil GL (1973). Cell-kill kinetics of several S-phase-specific drugs. *Cancer Research*.

[47] Sinclair WK (1965). Hydroxyurea: differential lethal effects on cultured mammalian cells during the cell cycle. *Science*.

[48] Sinclair WK (1967). Hydroxyurea: effects on Chinese hamster cells grown in culture. *Cancer Research*.

[49] Graham FL, Whitmore GF (1970). The effect of-beta-D-arabinofuranosylcytosine on growth, viability, and DNA synthesis of mouse L-cells. *Cancer Research*.

[50] Patwardhan B, Warude D, Pushpagandhan P, Bhatta N (2005). Ayurveda and traditional Chinese medicine: a comparative overview. *Evidence-Based Complementary and Alternative Medicine*.

